# Migrant mortality differences in the 2000s in Belgium: interaction with gender and the role of socioeconomic position

**DOI:** 10.1186/s12939-019-0983-5

**Published:** 2019-06-20

**Authors:** Katrien Vanthomme, Hadewijch Vandenheede

**Affiliations:** Interface Demography, Department of Social Research, Faculty of Economic and Social Sciences & Solvay Business School, Vrije Universiteit Brussel, Pleinlaan 2, 1050 Brussels, Belgium

**Keywords:** Belgium, Immigrants, Mortality, Inequalities, Socioeconomic position, Gender

## Abstract

**Background:**

Belgium has a long history of migration. As the migrant population is ageing, it is crucial thoroughly to document their health. Many studies that have assessed this, observed a migrant mortality advantage. This study will extend the knowledge by probing into the interaction between migrant mortality and gender, and to assess the role of socioeconomic position indicators in this paradox.

**Methods:**

Individually linked data of the 2001 Belgian Census, the National Register and death certificates for 2001–2011 were used. Migrant origin was based on both own and parents’ origin roots. We included native Belgians and migrants from the largest migrant groups aged 25 to 65 years. Absolute and relative mortality differences by migrant origin were calculated for the most common causes of death. Moreover, the Poisson models were adjusted for educational attainment, home ownership and employment status.

**Results:**

We observed a migrant mortality advantage for most causes of death and migrant groups, which was strongest among men. Adjusting for socioeconomic position generally increased the migrant mortality advantage, however with large differences by gender, migrant origin, socioeconomic position indicator and causes of death.

**Conclusions:**

Adjusting for socioeconomic position even accentuated the migrant mortality advantage although the impact varied by causes of death, migrant origin and gender. This highlights the importance of including multiple socioeconomic position indicators when studying mortality inequalities. Future studies should unravel morbidity patterns too since lower mortality not necessarily implies better health. The observed migrant mortality advantage suggests there is room for improvement. However, it is essential to organize preventative and curative healthcare that is equally accessible across social and cultural strata.

## Background

Many studies have already assessed the health status of migrant communities. These studies often show a migrant mortality paradox, at least among first-generation (FG) migrants [1–8]. Compared to the native population, FG migrants seem to have a mortality advantage which is quite paradoxical as they tend to belong to the lower socioeconomic strata of society [2, 5]. In literature, apart from being the result of possible data artefacts [1, 8], this paradox is explained in several ways. Firstly, to be able to cope with the challenges of the journey of migration, migrants are a selective group of healthy people, i.e. the *healthy migrant effect* [1, 5–12]. Secondly, migrants can take advantage of the healthy lifestyle of the home country [1, 5–8, 11] and of the well-developed and efficient healthcare system of the host country [1, 7, 12, 13]. In this way migrants combine the good things of the home and host country, leading to a *double advantage*. Migrants not only combine the advantages of both worlds, they also have been confronted with different exposures in both the home and host country [4, 9, 14, 15]. This could imply that FG migrants pass through the epidemiological transition at an accelerated pace, at least for migrants from non- or less industrialised countries [8, 15, 16]. The shift from unfavourable hygienic conditions in the home country to better hygienic conditions in the host country could lead to a decline in infectious-related mortality. At the same time migrants may adopt a western lifestyle which could increase chronic-disease mortality, yet only after a certain time lag; the so-called *migration-as-rapid-health-transition* hypothesis.

Studying cause-specific mortality patterns of the migrant population relative to that of the host country population may reveal important clues on disease aetiology and on the different hypotheses concerning migrant mortality [1, 9, 17, 18]. Belgium is a particularly apt setting to study this as it has a long history of migration [1, 2, 4], with nowadays a proportion of people with a migration background of about 20% [19]. As previous research has already probed this question [1, 4, 18], we will go a step further by adding gender and socioeconomic position (SEP) into the picture. Therefore, the objectives of this paper are to assess whether there is indeed a migrant mortality paradox in Belgium, to probe into the interaction with gender, and to assess the role of different SEP indicators in this paradox. More specifically, we will document migrant mortality patterns from 2001 to 2011 for all important migrant groups in Belgium for the five most important causes of death (COD). Since men and women have a different migration history [5, 20] we will focus on the interaction between migrant origin and gender in the analyses. Additionally, as life chances may be very different for migrants, as well as for migrant men and women, we will focus on the role of different SEP indicators in explaining these mortality differences.

## Data and methods

Data were derived from the individual linkage of three data sources: the Belgian census, the Belgian National Register and death certificates. In a first stage, a link was made by Statistics Belgium between the Belgian census of October 1st, 2001 and Register data for the follow-up period of 2001–2011. The census contains demographic and socioeconomic information for the total de jure population living in Belgium. The linkage with the National Register enabled us to account for all emigration and mortality during the study period. For all Belgian residents that died during the study period, cause-specific mortality was added by individual linkage with death certificates. The study population consisted of all Belgian residents with an attained age during follow-up between 25 and 64 years. The lower age limit was set to include a sufficient number of persons and deaths. Furthermore, the lower and upper age limit were set to be able to assess the various SEP indicators. For example when including employment status, it is essential to exclude persons outside the economically age range.

In this paper, our aim is to study the interaction between migrant mortality and gender, as well as the contribution of SEP to this association. More specifically, we intended to compare the mortality patterns of the native Belgian population with those of the largest migrant groups in Belgium, i.e. migrants originating from the neighbouring countries (the Netherlands (i.e. Dutch migrants) and France), Spain, Italy, Eastern Europe (Poland, Hungary, Romania, Czechoslovakia, Bulgaria, Czech Republic and Slovakia), Turkey, Morocco and Sub-Saharan Africa (SSA) (Congo (Zaire), Burundi and Rwanda). A migrant was defined using a stepwise method, based on both the individual and his/her parents’ country of origin, hereby maximizing the population with migrant roots. If an individual could be linked to his/her parents at the census of 2001, and one of his/her parents had its roots (i.e. nationality at birth) outside Belgium, we used this country as the individual’s country of origin. If the individual could not be linked to his/her parents in 2001, we checked whether the individual could be linked to his/her parents in the 1991 census, and then repeated the first two steps. If, however both parents had their origins in Belgium or if the parents’ nationality at birth was missing, the individual’s nationality at birth was used to define the individual’s country of origin. If this information was also lacking, current nationality was used to define the person’s country of origin. We did not distinguish first from second-generation migrants since previous research has already examined the different mortality patterns for both migrant generations in Belgium [18, 21, 22]. These studies generally observed a clear mortality advantage among first-generation migrants whereas the mortality patterns among second-generation migrants tended to converge towards mortality levels of native Belgians with similar SEP. Instead, in this paper we wanted to study the association with gender and SEP, two understudied subtopics in migrant mortality studies. We included three SEP indicators as they may represent different forms of disadvantage during different periods of life [23], and may have a different meaning in explaining mortality inequalities for men and women, migrant origin or COD. Educational attainment reflects chances early in life and may therefore not be the best suitable indicator among migrants [24, 25]. Employment status on the other hand is an SEP indicator at middle-age. Yet, given the different migration motivations of men and women, this indicator may be more informative for men than for women. That is why we also included home ownership, which refers to the economic assets at a household level. Educational attainment was categorized according to the International Standard Classification of Education (ISCED): primary education or no diploma (ISCED 0–1), lower secondary education (ISCED 2), upper secondary education (ISCED 3–4), and tertiary education (ISCED 5–6). Home ownership differentiates between tenants and owners of a dwelling. Finally, employment status contrasts employed people, unemployed people who are actively looking for a job, and unemployed people who are not looking for a job (e.g. students or retired people). The associations between migrant origin, gender and SEP were studied for all-cause mortality as well as for the five most common COD among Belgian adults, as classified according to the International Statistical Classification of Diseases and Related Health Problems (ICD - tenth revision): all cancers (C00-C99); circulatory diseases (I00-I99); lung cancer (C33–34); injuries (S00-T99); and respiratory diseases (J00-J99). Age-standardized mortality rates were calculated by gender and migrant origin as well as age-adjusted mortality rate ratios using age-adjusted Poisson models with native Belgians as the reference group. In these Poisson models, the interaction between migrant origin and gender was introduced. Furthermore, to assess the role of various indicators of SEP in these mortality differences, we adjusted the Poisson models for the three indicators of SEP: educational attainment (model 1), home ownership (model 2) and employment status (model 3). All analyses were performed using Stata/MP 14.2.

## Results

### Description of the migrant population

Belgium has a diverse migrant population. Roughly we can distinguish between migrants from the neighbouring countries (the Netherlands and France); migrants from traditional labour migration countries (Turkey, Morocco, Italy and to some extent Spain); and more recent migration influxes (from Sub-Saharan Africa, Eastern Europe and to some extent Spain).

In Table 1 the results of the descriptive analyses of the variables of interest are presented by migrant origin and gender. Migrant men and women from Western European countries were on average a bit older than the other migrant groups. Migrants of Dutch, Spanish and SSA descent were more highly educated compared with native Belgian men and women, while Italian, Turkish and Moroccan migrant men and women were less educated compared with their Belgian counterparts. While three out of four native Belgians were owners of a dwelling, this was far less the case for migrants. Especially among French, Spanish, Eastern European, Moroccan and SSA migrants of whom about half lived in a rented dwelling. The migrant population was more often unemployed compared with native Belgians. The level of unemployment was especially high among migrants from Turkey, Morocco, Italy, SSA and Eastern Europe. Yet, there was a noticeable difference between men and women with much higher levels of unemployment among migrant women, and high levels of unemployed but job seekers among migrant men.

**Table 1:** Description of the population by migrant origin and gender - Belgian residents aged 25–65 years, 2001–2011

		Number	Mean age	Education (%)				Ownership (%)		Employment status (%)		
				Primary	Lower sec.	Upper sec.	Tertiary	Owner	Tenant	Employed	Looking for job	Not looking for job
Belgium	Men	2,280,006	43.82	13.58	25.66	31.81	28.95	76.27	23.73	78.34	4.32	17.34
	Women	2,273,845	44.08	14.61	24.59	30.27	30.53	76.06	23.94	60.61	6.94	32.45
Netherlands	Men	46,459	42.83	10.64	22.73	32.90	33.73	69.08	30.92	81.21	3.50	15.29
	Women	42,556	42.88	12.48	26.26	31.12	30.14	69.71	30.29	56.97	6.71	36.32
France	Men	59,278	40.68	17.34	26.14	28.33	28.19	57.52	42.48	75.49	9.44	15.07
	Women	61,990	41.18	18.59	24.48	26.91	30.02	57.46	42.54	53.96	13.85	32.19
Spain	Men	11,861	42.86	8.42	14.22	23.03	54.33	51.35	48.65	84.41	6.08	9.51
	Women	10,446	41.91	7.98	15.48	27.62	48.91	53.73	46.27	58.87	8.16	32.97
Italy	Men	109,009	42.10	22.22	32.47	29.30	16.01	73.62	26.38	70.81	10.38	18.81
	Women	95,323	41.99	23.02	29.81	28.75	18.42	72.64	27.36	49.22	16.16	34.62
Eastern Europe	Men	29,084	40.98	13.64	25.82	32.16	28.39	53.87	46.13	68.18	13.64	18.18
	Women	34,098	40.46	14.36	22.66	29.01	33.97	53.42	46.58	46.90	18.98	34.13
Turkey	Men	33,575	37.92	39.34	26.22	26.06	8.38	63.94	36.06	55.95	20.80	23.26
	Women	29,959	38.00	53.58	20.94	19.53	5.95	66.68	33.32	23.68	22.36	53.95
Morocco	Men	62,756	38.68	32.94	22.48	27.39	17.20	43.20	56.80	56.88	22.24	20.88
	Women	51,301	38.37	44.99	20.77	22.76	11.48	47.08	52.92	27.73	20.61	51.66
SSA	Men	20,075	37.91	7.83	11.62	27.13	53.42	27.89	72.11	60.35	26.66	12.99
	Women	20,795	37.44	17.40	19.92	29.84	32.84	34.32	65.68	43.94	28.77	27.29

### Absolute and relative overall and cause-specific mortality inequalities by migrant origin and gender

In general, migrants had an overall absolute and relative mortality advantage compared with native Belgians (Tables 2, 3, 4, 5, 6, 7 and 8). Moroccan men and women particularly had lower overall mortality compared with native Belgians. For instance, during the study period 474.5 native Belgian men per 100,000 person-years died compared with 338.0 Moroccan men per 100,000 person-years (Table 2).

**Table 2:** Age-standardized mortality rates per 100,000 person-years by migrant origin, gender and cause of death - Belgian residents aged 25–65 years, 2001–2011

	All deaths	Cancer	Circulatory diseases	Lung cancer	Injuries	Respiratory diseases
Belgian men	474.5 (472.0–476.9)	175.5 (174.0–177.0)	107.2 (106.0–108.4)	63.7 (62.8–64.6)	37.1 (36.4–37.8)	31.6 (31.0–32.3)
Belgian women	254.7 (253.0–256.5)	116.4 (115.2–117.6)	45.1 (44.3–45.8)	21.6 (21.0–22.1)	15.0 (14.6–15.4)	15.4 (15.0–15.9)
Dutch men	360.1 (344.1–376.0)	143.0 (132.9–153.1)	78.7 (71.1–86.2)	48.0 (42.1–53.9)	33.8 (29.0–38.6)	18.8 (15.1–22.5)
Dutch women	220.0 (207.1–232.8)	107.1 (98.1–116.1)	36.3 (31.1–41.5)	26.4 (21.9–31.0)	12.0 (9.0–15.0)	15.0 (11.6–18.3)
French men	590.6 (570.2–611.1)	212.8 (200.4–225.3)	109.9 (100.9–118.9)	81.1 (73.4–88.8)	23.5 (19.7–27.3)	40.5 (34.9–46.1)
French women	303.9 (290.5–317.3)	126.8 (118.1–135.5)	45.7 (40.5–51.0)	25.4 (21.5–29.3)	10.6 (8.1–13.0)	15.5 (12.4–18.5)
Spanish men	338.7 (302.6–374.8)	137.6 (114.3–160.8)	54.2 (39.4–69.0)	41.4 (28.2–54.6)	19.2 (11.1–27.3)	17.4 (9.5–25.3)
Spanish women	205.7 (175.1–236.3)	90.3 (70.9–109.8)	22.7 (12.6–32.9)	14.5 (6.8–22.2)	16.5 (8.3–24.6)	16.9 (7.3–26.5)
Italian men	404.8 (393.4–416.2)	153.3 (146.2–160.4)	88.9 (83.5–94.2)	59.3 (54.8–63.7)	8.2 (6.7–9.8)	27.8 (24.7–30.9)
Italian women	216.1 (207.4–224.8)	96.8 (90.9–102.6)	42.1 (38.2–46.0)	15.0 (12.7–17.3)	3.3 (2.3–4.4)	12.1 (10.0–14.2)
East. Eur. men	515.8 (490.1–541.5)	172.1 (157.0–187.1)	115.9 (103.5–128.2)	70.1 (60.4–79.8)	25.6 (20.1–31.0)	29.8 (23.5–36.1)
East. Eur. women	263.7 (246.4–281.0)	113.1 (101.8–124.5)	45.2 (37.9–52.5)	22.6 (17.6–27.7)	13.3 (9.6–17.1)	12.9 (9.0–16.9)
Turkish men	440.0 (411.7–468.3)	128.2 (112.5–143.9)	98.7 (85.2–112.3)	55.4 (45.1–65.7)	23.7 (18.6–28.9)	29.1 (21.7–36.6)
Turkish women	249.2 (227.6–270.7)	70.3 (59.2–81.5)	57.2 (46.6–67.8)	8.6 (4.5–12.6)	10.4 (6.5–14.3)	13.3 (8.1–18.5)
Moroccan men	338.0 (322.0–353.9)	121.4 (111.6–131.2)	67.0 (59.8–74.2)	53.9 (47.3–60.5)	22.3 (18.7–25.8)	18.1 (14.3–21.9)
Moroccan women	214.0 (199.4–228.7)	71.9 (63.6–80.2)	44.0 (37.1–51.0)	6.7 (4.1–9.2)	9.7 (7.0–12.3)	12.3 (8.6–15.9)
SSA men	495.3 (444.4–546.1)	132.4 (105.1–159.7)	126.0 (98.7–153.4)	27.3 (14.1–40.4)	22.9 (15.4–30.4)	27.7 (14.8–40.7)
SSA women	293.5 (259.4–327.6)	105.2 (83.0–127.3)	50.7 (36.4–65.0)	14.1 (6.2–22.1)	10.6 (5.8–15.3)	13.2 (6.0–20.5)

**Table 3:** Relative all-cause mortality inequalities by migrant origin and gender, adjusted for age and SEP indicators - Belgian residents aged 25–65 years, 2001–2011

All deaths	Model 1		Model 2		Model 3		Model 4	
	MRR	95% C.I.	MRR	95% C.I.	MRR	95% C.I.	MRR	95% C.I.
Current age	1.09	1.09,1.09	1.08	1.08,1.08	1.09	1.09,1.09	1.07	1.07,1.07
Gender								
Men	(ref)		(ref)		(ref)		(ref)	
Women	0.54	0.53,0.54	0.53	0.53,0.54	0.54	0.53,0.54	0.43	0.42,0.43
Migrant origin								
Belgian	(ref)		(ref)		(ref)		(ref)	
Dutch	0.77	0.75,0.80	0.81	0.78,0.84	0.75	0.72,0.77	0.79	0.77,0.82
French	1.28	1.24,1.31	1.16	1.13,1.20	1.15	1.12,1.18	1.18	1.15,1.22
Spanish	0.77	0.71,0.83	0.88	0.81,0.96	0.70	0.64,0.76	0.87	0.80,0.94
Italian	0.82	0.80,0.84	0.73	0.71,0.74	0.82	0.80,0.84	0.73	0.71,0.75
East. Eur.	1.10	1.05,1.14	1.09	1.04,1.13	0.96	0.92,1.00	0.95	0.92,0.99
Turkish	0.79	0.76,0.83	0.61	0.58,0.64	0.73	0.69,0.77	0.56	0.53,0.58
Moroccan	0.70	0.68,0.73	0.55	0.53,0.57	0.57	0.55,0.59	0.50	0.48,0.52
SSA	0.90	0.85,0.96	1.09	1.02,1.16	0.70	0.66,0.75	0.76	0.71,0.81
Migrant origin X gender								
Belgian women	(ref)		(ref)		(ref)		(ref)	
Dutch women	1.14	1.07,1.21	1.10	1.03,1.16	1.16	1.09,1.23	1.06	1.00,1.13
French women	0.95	0.91,1.00	0.98	0.93,1.02	0.94	0.90,0.98	0.96	0.91,1.00
Spanish women	1.16	1.02,1.32	1.17	1.02,1.34	1.16	1.01,1.33	1.06	0.93,1.22
Italian women	1.01	0.97,1.05	0.99	0.95,1.03	1.02	0.98,1.06	1.01	0.97,1.05
East. Eur. Women	0.92	0.86,0.98	0.91	0.85,0.97	0.93	0.87,0.99	0.94	0.88,1.00
Turkish women	1.03	0.95,1.11	0.99	0.91,1.08	1.08	1.00,1.18	0.99	0.91,1.08
Moroccan women	1.05	0.99,1.12	1.03	0.97,1.11	1.11	1.04,1.18	1.07	1.00,1.14
SSA women	1.29	1.18,1.41	1.02	0.93,1.13	1.31	1.19,1.44	1.28	1.16,1.41
Educational attainment								
Primary or less			(ref)					
Lower secondary			0.73	0.73,0.74				
Upper secondary			0.58	0.58,0.59				
Tertiary			0.42	0.42,0.42				
Home ownership								
Owner					(ref)			
Tenant					1.93	1.92,1.94		
Employment status								
Employed							(ref)	
Unemployed and looking							2.35	2.32,2.38
Unemployed and not looking							2.75	2.73,2.77

Model 1 adjusted for current age; Model 2 adjusted for current age and educational attainment; Model 3 adjusted for current age and home ownership; Model 4 adjusted for current age and employment status

**Table 4:** Relative cancer mortality inequalities by migrant origin and gender, adjusted for age and SEP indicators - Belgian residents aged 25–65 years, 2001–2011

All cancers	Model 1		Model 2		Model 3		Model 4	
	MRR	95% C.I.	MRR	95% C.I.	MRR	95% C.I.	MRR	95% C.I.
Current age	1.12	1.12,1.12	1.11	1.11,1.11	1.12	1.12,1.12	1.11	1.11,1.11
Gender								
Men	(ref)		(ref)		(ref)		(ref)	
Women	0.67	0.66,0.68	0.67	0.66,0.68	0.67	0.67,0.68	0.61	0.60,0.62
Migrant origin								
Belgian	(ref)		(ref)		(ref)		(ref)	
Dutch	0.83	0.78,0.87	0.86	0.81,0.91	0.81	0.77,0.86	0.85	0.80,0.90
French	1.28	1.23,1.34	1.17	1.12,1.23	1.19	1.13,1.24	1.22	1.16,1.27
Spanish	0.88	0.78,0.99	1.01	0.89,1.14	0.85	0.75,0.97	0.99	0.88,1.12
Italian	0.86	0.83,0.89	0.80	0.77,0.83	0.86	0.83,0.90	0.81	0.78,0.85
East. Eur.	0.99	0.92,1.06	0.98	0.91,1.05	0.89	0.82,0.95	0.93	0.86,1.00
Turkish	0.71	0.65,0.77	0.63	0.57,0.69	0.68	0.62,0.74	0.60	0.55,0.66
Moroccan	0.71	0.67,0.76	0.60	0.56,0.64	0.61	0.57,0.65	0.58	0.55,0.62
SSA	0.71	0.63,0.81	0.87	0.77,0.99	0.61	0.54,0.70	0.68	0.60,0.77
Migrant origin X gender								
Belgian women	(ref)		(ref)		(ref)		(ref)	
Dutch women	1.12	1.03,1.22	1.08	0.99,1.19	1.13	1.04,1.24	1.09	1.00,1.19
French women	0.89	0.83,0.96	0.96	0.89,1.03	0.87	0.81,0.94	0.91	0.84,0.97
Spanish women	1.13	0.93,1.37	1.06	0.86,1.30	1.08	0.88,1.32	1.00	0.82,1.22
Italian women	1.00	0.94,1.06	0.99	0.93,1.06	1.00	0.94,1.06	0.98	0.92,1.04
East. Eur. Women	1.02	0.92,1.13	1.02	0.91,1.14	1.03	0.92,1.15	1.02	0.91,1.13
Turkish women	0.93	0.80,1.07	0.89	0.76,1.04	0.94	0.81,1.09	0.89	0.76,1.03
Moroccan women	0.96	0.87,1.06	1.02	0.91,1.13	0.98	0.88,1.09	1.01	0.90,1.12
SSA women	1.24	1.04,1.48	1.02	0.85,1.23	1.18	0.98,1.43	1.16	0.97,1.40
Educational attainment								
Primary or less			(ref)					
Lower secondary			0.86	0.85,0.87				
Upper secondary			0.75	0.74,0.76				
Tertiary			0.59	0.58,0.60				
Home ownership								
Owner					(ref)			
Tenant					1.64	1.62,1.66		
Employment status								
Employed							(ref)	
Unemployed and looking							1.77	1.73,1.81
Unemployed and not looking							1.57	1.55,1.59

Model 1 adjusted for current age; Model 2 adjusted for current age and educational attainment; Model 3 adjusted for current age and home ownership; Model 4 adjusted for current age and employment status

**Table 5:** Relative circulatory diseases mortality inequalities by migrant origin and gender, adjusted for age and SEP indicators - Belgian residents aged 25–65 years, 2001–2011

Circulatory	Model 1		Model 2		Model 3		Model 4	
	MRR	95% C.I.	MRR	95% C.I.	MRR	95% C.I.	MRR	95% C.I.
Current age	1.12	1.12,1.12	1.11	1.11,1.12	1.13	1.13,1.13	1.10	1.10,1.10
Gender								
Men	(ref)		(ref)		(ref)		(ref)	
Women	0.41	0.40,0.41	0.39	0.39,0.40	0.40	0.39,0.41	0.33	0.33,0.34
Migrant origin								
Belgian	(ref)		(ref)		(ref)		(ref)	
Dutch	0.75	0.69,0.80	0.78	0.72,0.84	0.71	0.66,0.77	0.75	0.69,0.81
French	1.09	1.03,1.16	0.98	0.92,1.05	0.96	0.90,1.03	1.02	0.96,1.09
Spanish	0.58	0.48,0.70	0.67	0.54,0.83	0.52	0.42,0.64	0.67	0.55,0.82
Italian	0.85	0.81,0.89	0.76	0.72,0.80	0.85	0.81,0.89	0.76	0.72,0.80
East. Eur.	1.14	1.05,1.24	1.12	1.03,1.23	0.96	0.88,1.05	1.00	0.92,1.09
Turkish	0.91	0.82,1.01	0.69	0.62,0.78	0.83	0.74,0.92	0.65	0.58,0.72
Moroccan	0.63	0.58,0.69	0.52	0.48,0.58	0.51	0.46,0.55	0.48	0.44,0.52
SSA	1.04	0.91,1.18	1.28	1.11,1.48	0.76	0.66,0.88	0.87	0.76,1.01
Migrant origin X gender								
Belgian women	(ref)		(ref)		(ref)		(ref)	
Dutch women	1.12	0.98,1.29	1.09	0.95,1.27	1.16	1.01,1.34	1.08	0.93,1.24
French women	0.99	0.89,1.10	1.05	0.93,1.18	0.98	0.87,1.10	1.01	0.90,1.13
Spanish women	1.06	0.74,1.53	1.15	0.78,1.69	1.10	0.74,1.62	1.02	0.70,1.49
Italian women	1.08	0.99,1.18	1.10	1.00,1.21	1.12	1.02,1.22	1.11	1.02,1.22
East. Eur. Women	0.88	0.76,1.02	0.85	0.72,1.00	0.89	0.76,1.04	0.84	0.71,0.99
Turkish women	1.28	1.07,1.52	1.20	0.99,1.46	1.32	1.10,1.59	1.28	1.06,1.55
Moroccan women	1.36	1.17,1.57	1.30	1.11,1.53	1.43	1.22,1.67	1.33	1.13,1.56
SSA women	1.34	1.08,1.66	1.05	0.83,1.34	1.34	1.06,1.71	1.33	1.05,1.68
Educational attainment								
Primary or less			(ref)					
Lower secondary			0.77	0.76,0.78				
Upper secondary			0.63	0.62,0.64				
Tertiary			0.40	0.39,0.41				
Home ownership								
Owner					(ref)			
Tenant					2.17	2.14,2.21		
Employment status								
Employed							(ref)	
Unemployed and looking							2.36	2.29,2.43
Unemployed and not looking							2.40	2.35,2.44

Model 1 adjusted for current age; Model 2 adjusted for current age and educational attainment; Model 3 adjusted for current age and home ownership; Model 4 adjusted for current age and employment status

**Table 6:** Relative lung cancer mortality inequalities by migrant origin and gender, adjusted for age and SEP indicators - Belgian residents aged 25–65 years, 2001–2011

Lung cancer	Model 1		Model 2		Model 3		Model 4	
	MRR	95% C.I.	MRR	95% C.I.	MRR	95% C.I.	MRR	95% C.I.
Current age	1.13	1.13,1.13	1.12	1.12,1.12	1.14	1.13,1.14	1.12	1.12,1.12
Gender								
Men	(ref)		(ref)		(ref)		(ref)	
Women	0.36	0.35,0.36	0.35	0.34,0.36	0.36	0.35,0.36	0.32	0.31,0.33
Migrant origin								
Belgian	(ref)		(ref)		(ref)		(ref)	
Dutch	0.72	0.65,0.80	0.76	0.69,0.85	0.71	0.64,0.79	0.75	0.68,0.83
French	1.34	1.25,1.44	1.18	1.08,1.28	1.21	1.12,1.31	1.26	1.17,1.36
Spanish	0.72	0.58,0.90	0.85	0.67,1.09	0.67	0.53,0.84	0.82	0.65,1.03
Italian	0.94	0.89,1.00	0.82	0.77,0.87	0.95	0.89,1.01	0.88	0.83,0.93
East. Eur.	1.10	0.98,1.22	1.07	0.95,1.21	0.95	0.85,1.07	1.02	0.91,1.14
Turkish	0.86	0.75,0.98	0.67	0.57,0.77	0.80	0.69,0.92	0.69	0.60,0.80
Moroccan	0.87	0.80,0.96	0.63	0.57,0.70	0.70	0.63,0.77	0.68	0.62,0.75
SSA	0.35	0.26,0.47	0.48	0.36,0.66	0.29	0.21,0.39	0.33	0.25,0.45
Migrant origin X gender								
Belgian women	(ref)		(ref)		(ref)		(ref)	
Dutch women	1.63	1.38,1.92	1.54	1.29,1.84	1.64	1.39,1.94	1.54	1.30,1.83
French women	0.94	0.82,1.07	1.05	0.91,1.22	0.91	0.79,1.05	0.95	0.83,1.10
Spanish women	1.03	0.66,1.62	1.00	0.61,1.63	1.02	0.63,1.65	0.86	0.53,1.40
Italian women	0.77	0.67,0.87	0.80	0.70,0.91	0.78	0.68,0.89	0.75	0.65,0.86
East. Eur. Women	0.95	0.78,1.17	0.99	0.80,1.24	0.97	0.78,1.20	0.94	0.76,1.16
Turkish women	0.37	0.25,0.55	0.26	0.16,0.42	0.35	0.23,0.54	0.38	0.25,0.58
Moroccan women	0.37	0.28,0.49	0.42	0.31,0.58	0.34	0.25,0.47	0.43	0.32,0.58
SSA women	1.92	1.22,3.01	1.41	0.87,2.29	2.15	1.35,3.41	1.93	1.21,3.07
Educational attainment								
Primary or less			(ref)					
Lower secondary			0.77	0.75,0.79				
Upper secondary			0.58	0.56,0.59				
Tertiary			0.36	0.34,0.37				
Home ownership								
Owner					(ref)			
Tenant					2.05	2.01,2.09		
Employment status								
Employed							(ref)	
Unemployed and looking							2.10	2.02,2.18
Unemployed and not looking							1.67	1.63,1.71

Model 1 adjusted for current age; Model 2 adjusted for current age and educational attainment; Model 3 adjusted for current age and home ownership; Model 4 adjusted for current age and employment status

**Table 7:** Relative respiratory diseases mortality inequalities by migrant origin and gender, adjusted for age and SEP indicators - Belgian residents aged 25–65 years, 2001–2011

Respiratory	Model 1		Model 2		Model 3		Model 4	
	MRR	95% C.I.	MRR	95% C.I.	MRR	95% C.I.	MRR	95% C.I.
Current age	1.15	1.15,1.15	1.13	1.13,1.14	1.15	1.15,1.16	1.10	1.10,1.10
Gender								
Men	(ref)		(ref)		(ref)		(ref)	
Women	0.49	0.48,0.51	0.47	0.46,0.49	0.49	0.47,0.50	0.36	0.35,0.38
Migrant origin								
Belgian	(ref)		(ref)		(ref)		(ref)	
Dutch	0.59	0.50,0.69	0.65	0.55,0.77	0.54	0.45,0.64	0.62	0.52,0.73
French	1.39	1.25,1.54	1.29	1.15,1.45	1.16	1.03,1.30	1.32	1.18,1.47
Spanish	0.70	0.50,0.97	0.73	0.49,1.11	0.44	0.29,0.67	0.66	0.44,0.99
Italian	0.80	0.73,0.88	0.66	0.60,0.73	0.79	0.72,0.87	0.68	0.61,0.75
East. Eur.	0.97	0.82,1.15	1.02	0.85,1.23	0.81	0.68,0.97	0.88	0.74,1.05
Turkish	0.84	0.68,1.02	0.52	0.41,0.66	0.72	0.57,0.90	0.50	0.40,0.62
Moroccan	0.62	0.53,0.73	0.43	0.36,0.52	0.46	0.39,0.55	0.42	0.35,0.50
SSA	0.83	0.62,1.11	1.11	0.79,1.54	0.51	0.36,0.71	0.63	0.45,0.88
Migrant origin X gender								
Belgian women	(ref)		(ref)		(ref)		(ref)	
Dutch women	1.77	1.40,2.24	1.59	1.22,2.06	1.85	1.44,2.37	1.60	1.24,2.06
French women	0.79	0.66,0.95	0.66	0.53,0.82	0.76	0.62,0.93	0.73	0.59,0.89
Spanish women	1.60	0.95,2.69	2.37	1.34,4.21	2.10	1.13,3.88	1.84	1.01,3.32
Italian women	1.03	0.88,1.22	1.03	0.86,1.23	1.09	0.92,1.29	1.14	0.97,1.35
East. Eur. Women	0.84	0.62,1.12	0.88	0.64,1.20	0.84	0.62,1.15	0.84	0.62,1.14
Turkish women	0.83	0.57,1.22	0.76	0.48,1.20	0.94	0.63,1.39	0.84	0.55,1.29
Moroccan women	1.32	1.01,1.73	1.33	0.98,1.80	1.24	0.92,1.68	1.34	1.00,1.80
SSA women	1.55	1.01,2.39	1.17	0.72,1.91	1.54	0.92,2.55	1.67	1.04,2.69
Educational attainment								
Primary or less			(ref)					
Lower secondary			0.62	0.60,0.64				
Upper secondary			0.43	0.41,0.44				
Tertiary			0.24	0.23,0.25				
Home ownership								
Owner					(ref)			
Tenant					2.91	2.83,2.99		
Employment status								
Employed							(ref)	
Unemployed and looking							3.53	3.33,3.74
Unemployed and not looking							4.58	4.40,4.76

Model 1 adjusted for current age; Model 2 adjusted for current age and educational attainment; Model 3 adjusted for current age and home ownership; Model 4 adjusted for current age and employment status

**Table 8:** Relative injuries mortality inequalities by migrant origin and gender, adjusted for age and SEP indicators - Belgian residents aged 25–65 years, 2001–2011

Injuries	Model 1		Model 2		Model 3		Model 4	
	MRR	95% C.I.	MRR	95% C.I.	MRR	95% C.I.	MRR	95% C.I.
Current age	1.02	1.02,1.02	1.01	1.01,1.01	1.02	1.02,1.02	1.01	1.01,1.01
Gender								
Men	(ref)		(ref)		(ref)		(ref)	
Women	0.39	0.38,0.40	0.39	0.38,0.40	0.39	0.38,0.40	0.35	0.34,0.36
Migrant origin								
Belgian	(ref)		(ref)		(ref)		(ref)	
Dutch	0.98	0.88,1.08	0.98	0.88,1.09	0.95	0.85,1.06	0.98	0.88,1.09
French	0.68	0.61,0.76	0.60	0.53,0.68	0.62	0.55,0.69	0.59	0.52,0.67
Spanish	0.57	0.42,0.77	0.56	0.40,0.79	0.47	0.34,0.66	0.53	0.38,0.74
Italian	0.22	0.19,0.25	0.19	0.16,0.22	0.21	0.18,0.24	0.20	0.17,0.23
East. Eur.	0.74	0.64,0.86	0.70	0.59,0.82	0.67	0.58,0.78	0.66	0.56,0.77
Turkish	0.78	0.68,0.88	0.64	0.56,0.74	0.67	0.58,0.78	0.63	0.55,0.72
Moroccan	0.70	0.63,0.77	0.52	0.47,0.59	0.53	0.47,0.59	0.48	0.43,0.54
SSA	0.73	0.61,0.88	0.77	0.63,0.95	0.49	0.40,0.61	0.57	0.46,0.70
Migrant origin X gender								
Belgian women	(ref)		(ref)		(ref)		(ref)	
Dutch women	0.85	0.69,1.05	0.79	0.63,0.99	0.80	0.64,1.00	0.76	0.61,0.96
French women	1.04	0.85,1.26	1.04	0.83,1.30	0.94	0.76,1.17	1.03	0.83,1.28
Spanish women	2.11	1.34,3.32	2.65	1.63,4.31	2.45	1.50,4.00	2.51	1.55,4.06
Italian women	1.08	0.84,1.39	0.99	0.74,1.32	0.95	0.71,1.27	0.90	0.68,1.20
East. Eur. Women	1.15	0.90,1.47	1.05	0.79,1.39	1.15	0.89,1.49	1.08	0.83,1.42
Turkish women	0.88	0.68,1.14	0.82	0.62,1.09	1.02	0.78,1.34	0.86	0.66,1.14
Moroccan women	0.97	0.80,1.18	0.84	0.66,1.08	0.90	0.71,1.13	0.96	0.76,1.21
SSA women	1.08	0.79,1.49	1.02	0.72,1.46	1.39	0.97,1.97	1.17	0.82,1.68
Educational attainment								
Primary or less			(ref)					
Lower secondary			0.83	0.80,0.86				
Upper secondary			0.73	0.71,0.76				
Tertiary			0.48	0.46,0.50				
Home ownership								
Owner					(ref)			
Tenant					1.88	1.84,1.93		
Employment status								
Employed							(ref)	
Unemployed and looking							1.84	1.77,1.92
Unemployed and not looking							1.85	1.80,1.90

Model 1 adjusted for current age; Model 2 adjusted for current age and educational attainment; Model 3 adjusted for current age and home ownership; Model 4 adjusted for current age and employment status

In contrast, migrant men and women from French descent and migrant men from Eastern European descent had higher overall mortality compared with native Belgians. For instance, French men and women had respectively 28 and 22% (MRR_French women_ = 0.95 x MRR_French_ = 1.28) higher all-cause mortality compared with native Belgian men and women. When we look at the cause-specific mortality analyses, we observed similar findings as for all-cause mortality (Tables 2, 3, 4, 5, 6, 7 and 8). In general, there were three main findings: firstly, we observed a migrant mortality advantage for most COD and most migrant groups, with the exception of French men and women, and Eastern European men; secondly, mortality among men showed more variation by migrant background than among women. For instance, for mortality due to circulatory and respiratory diseases (Tables 5 and 7), there was no variation by migrant background among women, while among men Dutch, Spanish, Italian and Moroccan men had lower mortality compared with native Belgian men, and French and Eastern European men had elevated mortality rates as compared with native Belgian men. A third conclusion is that there is variation by COD as well. Among women, the largest mortality advantage was observed for Turkish and Moroccan women: for instance, for lung cancer, their mortality was 68% (MRR_Moroccanwomen_ = 0.37 x MRR_Moroccan_ = 0.87) lower compared with native Belgian women (Table 6), whereas for respiratory diseases mortality (Table 7), there was no mortality difference compared with Belgian women.

### The role of SEP in overall and cause-specific mortality differences by migrant origin and gender

In models 2 to 4, the association between migrant origin and (cause-specific) mortality has been adjusted for educational attainment, home ownership and employment status respectively (Tables 3, 4, 5, 6, 7 and 8).

The association between these SEP variables and cause-specific mortality was as expected: being highly educated, a homeowner, and employed resulted in lower all-cause and cause-specific mortality. In this paper, we were interested in what changes occurred to the mortality differences when the SEP variables were added to the models. As could be expected, based on the generally lower SE profile of the migrant groups, adjusting for the SEP variables generally increased the migrant mortality advantage. However, there were large differences by gender, migrant origin, SEP indicator and COD. The main conclusions are summarized below. Firstly, as mentioned before, adjusting for SEP generally led to an increase in the migrant mortality advantage. This happens in three ways: the existing mortality advantage increased; the migrant mortality disadvantage disappeared; the migrant mortality disadvantage transformed into a mortality advantage. To illustrate the first, we will use the example of all-cause mortality among Moroccan migrants: where Moroccan men and women had respectively 30 and 26% (MRR_Moroccan women_ = 1.05 x MRR_Moroccan_ = 0.70) lower all-cause mortality in the unadjusted model, accounting for employment status the mortality advantage increased to 50 and 46% (MRR_Moroccan women_ = 1.07 x MRR_Moroccan_ = 0.50) (Table 3). On the other hand, SSA women had an excess all-cause mortality of 16% (MRR_SSAwomen_ = 1.29 x MRR_SSA_ = 0.90) in the unadjusted model, while after adding the SEP variables, there was no longer a mortality difference as compared with Belgian women (Table 3). The third can be illustrated by all-cause mortality among Eastern European men (Table 3). In the unadjusted model, they had an excess mortality of 10% compared with native Belgian men. Adding ownership and employment status to the model resulted in a mortality advantage of about 5% compared with native Belgian men. Yet, there were some exceptions to this trend. The excess all-cause mortality among French men and women remained after adjustment for SEP (Table 3). For some groups, adjusting for SEP even had a negative effect on the mortality patterns. This was mainly the case among Spanish and SSA migrant men when educational attainment was included in the model. For instance, compared with Belgian men, Spanish men had a cancer mortality advantage of 12% in the unadjusted model, which disappeared when accounting for education and employment status (Table 4). Furthermore, the all-cause mortality advantage of SSA men was even reversed in a mortality disadvantage when adjusting for educational attainment (Table 3).

A second conclusion is that adding SEP to the models had a larger impact among men than among women. Most variation by migrant background was observed among men and most changes in mortality differences after SEP was taken into account occurred among men (Tables 3, 4, 5, 6, 7 and 8). Yet, where in men, including SEP sometimes resulted in a decrease of the mortality advantage, in women if changes occurred, these were always in favour of the migrant groups, with only one exception: accounting for home ownership explained the injuries mortality advantage among Turkish women (Table 8).

Thirdly, next to gender, we also observed large variation by migrant background, SEP indicator and COD. All in all, taking SEP into account more often affected the patterns for all-cause mortality and mortality from circulatory and respiratory diseases, while it had less impact on mortality from cancers and injuries. SEP did not seem to explain mortality differences between Belgians and Dutch migrants. Among French men and women, adding SEP had a positive effect on mortality differences. Yet, especially among men, excess mortality remained for all-cause mortality and cancer, lung cancer and respiratory mortality. Among French women, the excess mortality only remained for all-cause mortality (Table 3). Among Spanish migrants, the results were also different for men and women. Among Spanish women there were no mortality differences compared with Belgians, neither in the unadjusted, nor the adjusted model. Yet, among Spanish men, adding SEP (especially education and employment) resulted in no change or even a disadvantageous mortality pattern. Among Italian men, adding educational attainment and employment status led to an increase in the mortality advantage compared with native Belgians, while among Italian women, SEP did not seem to have an impact on the mortality patterns. Similar results were observed for Eastern European migrants: among women, adjusting for SEP resulted only in a mortality advantage for all-cause mortality, while among men, the impact was larger and resulted in a mortality advantage for all causes, cancer, circulatory, and respiratory diseases. Among Turkish and Moroccan migrant men and women, adding SEP to the models resulted in a larger mortality advantage. Especially adjusting for educational attainment and employment status had an effect. Finally, among SSA the impact of SEP was different for men and women as well as by indicator of SEP. Among women, adjusting for SEP (especially home ownership) explained the all-cause mortality disadvantage and even resulted in a cancer mortality advantage (Tables 3 and 4). However, among men, including home ownership and to a lesser extent employment status resulted in a mortality advantage for all-cause mortality, circulatory diseases, respiratory diseases and injuries. On the contrary, adjusting for education led to a mortality disadvantage for all-cause mortality and mortality from circulatory diseases.

## Discussion and conclusion

### Strengths and weaknesses

The results of this study are based upon an exhaustive nationwide dataset covering sociodemographic, socioeconomic, migration and mortality information for the total de jure population in the period 2001–2011. These variables are stemming from three data sources (the Census, the National Registry and the death certificates) which were individually linked by Statistics Belgium, hereby avoiding a numerator-denominator bias. We disposed of information on all legal residents in Belgium at the moment of the census (October 1st 2001), and whether or not they emigrate or die during follow-up. The data are left truncated, meaning that immigrants who arrived later than the census were not included in this study.

Additional migration information such as the reason for migration was not available in the dataset. We were able to include all important migrant groups in Belgium, thereby maximizing the population with migrant roots. In addition, as men and women have a different migration history [1, 2, 4, 6, 13, 20], we decided to focus on the interplay between migrant origin, gender, SEP and cause-specific mortality, which is a topic that is still understudied [26]. Men used to migrate for work purposes and therefore had to be in good health and were more likely to receive medical checks at the workplace. Women on the other hand migrated for family reasons and often did not work outside the home. This different trajectory may have implications both for their health and for their SEP, as we indeed observed in our results (see next paragraph). Another strength of this paper is that we included three different SEP indicators, all representing different forms of disadvantage during different periods in life [23], and clearly having a different meaning in explaining mortality inequalities for men and women, and by migrant origin or COD. However, these indicators also have limits, e.g.: employment status does not contain information on the type of job, the working regime or the job history. Similar for home ownership, which is a proxy for the economic wealth of the household, yet it does not include information on the type or quality of the home, or the household income. These additional indicators of SEP could also have been informative, but were not present in our dataset. Including inequalities in morbidity patterns could be informative as well about the pathways behind the observed mortality patterns but this information was not available. Similarly, due to the semi-closed nature of the dataset, we were not able to include exposures experienced during the life course, prior to the migration to Belgium. Neither did we obtain data on health behaviours or healthcare use, which are likely to be gender- and culture-specific [6, 27–29].

### Reflections on the main findings of the study

While describing the general characteristics of the various groups, we observed that the migrant community in Belgium is a very diverse group, as was stated in previous research as well [2]. Large variations have been observed for all variables of interest. In general terms we could discern different profiles. Migrants from Dutch descent were quite similar to Belgians in terms of their socioeconomic profile, while French migrants had a somewhat lower SEP compared with Belgians. Migrants from Italian, Turkish and Moroccan descent were those migrant groups that belonged to the early migration waves. They generally were situated in the lower socioeconomic strata. Migrants from Spanish descent are a diverse group including both traditional labour migrants as well as a recent influx of highly educated immigrants. Migrants from SSA descent belonged to the most recent influx of immigrants, and were more likely to be higher educated, yet living in rented dwellings. Furthermore, high levels of SSA migrants were unemployment but looking for a job. The last group, the migrants from Eastern European descent were a mixed group of traditional labour immigrants and more recent immigrants that were characterized by quite similar educational levels as the native Belgians, but higher levels of living in rented dwellings and unemployment. Finally, some important differences were found between men and women, indicating a different migration history and dependent on the country of origin. The fact that men most often migrated for job issues while women later followed for reasons of family reunification [2, 6, 20] was reflected in our results, at least among the earliest migrant groups. In these groups, women were most often unemployed at home while men were more often at work.

The unadjusted cause-specific mortality analyses by migrant origin confirmed the migrant mortality paradox for most migrant groups and all studied COD, as was also observed in previous studies [1, 4–8, 30]. Even though immigrants were more often situated in the lower socioeconomic strata, they tended to show advantageous mortality patterns. Among the different migrant origins, largest mortality advantages were found for Turkish and Moroccan migrants, without and with adjustment for SEP. This could be explained by the fact that compared with native Belgians, these traditional migrant groups may maintain a healthier lifestyle with earlier reproductive behaviour, longer breastfeeding periods, lower levels of alcohol and tobacco consumption, and a Mediterranean diet with high fruit and vegetable consumption [1, 6, 8, 9, 13, 17, 20, 26, 31–34]. Migrants from less industrialized countries thus seem to profit from the *double advantage* of maintaining the healthy lifestyle of the home country combined with the high-quality and accessible healthcare system of the host country [1, 7, 12, 13]. In contrast, migrants from French and to some extent Eastern European descent were the exceptions showing higher all-cause and cause-specific mortality compared with the native population. Again, part of the explanation is likely to be found in a difference in lifestyle as previous research has shown that these groups have high levels of tobacco and alcohol consumption [35]. For some part, the mortality disadvantage among French and in particular Eastern European migrants can be explained by their lower SEP, as in the adjusted models some of their excess mortality disappeared.

In general, we can say that men showed more variation in mortality by migrant origin than women, which we expected based on the *healthy migrant effect*. Besides the French and to some extent Eastern European migrant men, the mortality differences were generally in favour of the migrant groups. The fact that more differences were observed among men could be related to the differential history of migration: men used to migrate for job reasons and therefore had to be in good health [6, 13, 20]. Moreover, as men were more often employed, they were perhaps more likely to receive medical checks at work [13]. Women on the other hand migrated for family reasons, did not have a job but instead took care of the household, which did not necessarily demand good health. However, a counterargument is that the type of jobs offered to migrant men and women are different, implying different health risks [26, 36]: where women were more likely to be employed (if employed) in domestic or caretaking services, men were more likely to work in risky sectors as mining, agriculture or construction. On the other hand, not having a job and being dependent of their family or partner makes them even more vulnerable as they have a double disadvantage of being both a migrant and a women [26, 36]. However, if this was true, we would perhaps have observed a larger impact on the mortality differences among women when adjusting for SEP, which wasn’t the case. Instead, SEP seemed to have a larger impact on the mortality patterns of men than of women.

Adjusting the mortality differences for SEP generally resulted in an increased mortality advantage, although there was quite some variation by gender, COD and migrant origin. Among Turkish and Moroccan migrants, adjusting for the indicators of SEP even increased their mortality advantage compared with the native Belgian population. This did not come as a surprise since they belonged to the early migration flows who were more often situated in the lower SE strata. Among the other traditional early migration group, the Italians, adjustment for educational attainment and employment status resulted in an improvement of the mortality patterns whereas adjustment for ownership had no effect or a reverse effect. From the descriptive analyses we can deduce that people with an Italian background had similar levels of home ownership but lower levels of high education and employment, which could explain these results. Some of the migrant groups (Turkish, Moroccan and SSA) are on average younger, which could be related to this, as ownership increases with age. Among migrants from Eastern European descent, the mortality disadvantage disappeared after adjustment for SEP. On the contrary, among French migrants, the mortality disadvantage remained significant among men while among women the excess mortality had disappeared. Exceptions to these findings were Spanish and SSA men, whose mortality patterns even deteriorated after accounting for education. Striking however, no such change was observed among Spanish and SSA women. SSA men had an initial mortality advantage as a result of the protective effect of their educational level, while SSA women did not have a mortality advantage due to their high levels of unemployment.

As mentioned before, we observed quite some variation by indicator of SEP. There is not one indicator that was most important for all groups, yet the impact of the indicators of SEP varied by COD, migrant origin and gender. This stresses the importance of including SEP, and more specifically including more than one indicator of SEP when assessing mortality inequalities in the population. We expected that ownership would be a more important SEP marker for women as it reflects the financial means at the household level, including the earnings from the husband [37]. Since women are less likely to be employed and if employed have lower wages than men, we assumed this to be an important issue, certainly among the traditional migrant groups where employment levels are rather low. Yet, this was not reflected in our results, which could be due to the fact that we combined first- and second-generation migrants. The only main finding when it comes to indicator of SEP was that among men, and especially Spanish and SSA men adjusting for education had a negative impact on their mortality patterns, which was due to their high educational levels.

## Implications and conclusion

In order to provide high-quality, accessible and adequate healthcare, it is necessary continuously to monitor the mortality patterns of the population, including the ageing migrant population [1, 18, 23, 30]. Future studies should also take morbidity differences into account between the migrant and host population, since lower mortality not necessarily implies better health. Moreover, longitudinal studies, including information on life-long exposures, health behaviours and healthcare use might allow for a better understanding of the nature of health inequalities [15, 30].

We learned that migrants generally had lower mortality than the native Belgian population, for some groups even after adjustment for SEP. This suggests that there is potential room for improvement to protect our health [1, 18]. Primary prevention aiming at the adaptation of the typical western lifestyle with high levels of consumption of tobacco, alcohol and meat and a sedentary lifestyle could have a huge effect on the mortality pattern in countries such as Belgium [8, 18, 23, 30, 38], as well as in other European countries, taking the example of the high lifestyle-related mortality among French migrants.

This study stressed the importance of integrating a gender perspective in studies assessing mortality and morbidity patterns within the migrant and host population [26]. We observed larger migrant mortality variation among men. Yet, family healthcare is often organized by the women in immigrant families, and these women are more often unemployed and hence at risk of being socially isolated. Therefore, it is essential to eliminate structural, social, financial and cultural barriers for healthcare use by migrant men and women [26, 39, 40]. Since SEP had quite some impact on the mortality differentials, organising preventative and curative healthcare that is equally accessible across social strata is also important [26]. Our results suggest that one’s SEP may contribute as a protective factor and vice versa. It is thus essential to stimulate good housing and employment chances among all social strata and migrant groups [4, 18], not only for their well-being but also in terms of physical health.

## Data Availability

Data are from a census-linked mortality follow-up study and cannot be made available due to privacy issues. Researchers can gain full access to the data by submitting an application to the Privacy Commission Belgium. In order to get permission to use data from the Belgian population register linked to census data an authorization request (in Dutch or French) needs to be submitted to the Belgian Data Protection Authority. The authorization request includes an application form and additional forms regarding data security. The necessary forms for the authorization request can be downloaded from the Data Protection Authority website (https://www.dataprotectionauthority.be). ﻿Next toinformation on the applicant and a list of requested data, the authorization request should specify why the data from the population register are necessary, for which time span data will be stored, and who will have access to the data.

## Abbreviations

COD: Causes of death
FG: First-generation
ICD: International Statistical Classification of Diseases and Related Health Problems
ISCED: International Standard Classification of Education
SEP: Socioeconomic position
SSA: Sub-Saharan Africa
